# Heparin and Liver Heparan Sulfate Can Rescue Hepatoma Cells from Topotecan Action

**DOI:** 10.1155/2014/765794

**Published:** 2014-09-07

**Authors:** József Dudás, József Bocsi, Alexandra Fullár, Kornélia Baghy, Tibor Füle, Saule Kudaibergenova, Ilona Kovalszky

**Affiliations:** ^1^Department Otorhinolaryngology, Medical University Innsbruck, Anichstrasse 35, 6020 Innsbruck, Austria; ^2^First Institute of Pathology & Experimental Cancer Research, Faculty of Medicine, Semmelweis University, Üllöi út 26, 1085 Budapest, Hungary; ^3^Department of Otorhinolaryngology, Faculty Medical Department, Asfendiyarov Kazakh National Medical University, Specialty Otorhinolaryngology, Index 05-00-12, Almaty, Kazakhstan

## Abstract

Topotecan (TpT) is a major inhibitory compound of topoisomerase (topo) I that plays important roles in gene transcription and cell division. We have previously reported that heparin and heparan sulfate (HS) might be transported to the cell nucleus and they can interact with topoisomerase I. We hypothesized that heparin and HS might interfere with the action of TpT. To test this hypothesis we isolated topoisomerase I containing cell nuclear protein fractions from normal liver, liver cancer tissues, and hepatoma cell lines. The enzymatic activity of these extracts was measured in the presence of heparin, liver HS, and liver cancer HS. In addition, topo I activity, cell viability, and apoptosis of HepG2 and Hep3B cells were investigated after heparin and TpT treatments. Liver cancer HS inhibited topo I activity in vitro. Heparin treatment abrogated topo I enzyme activity in Hep3B cells, but not in HepG2 cells, where the basal activity was higher. Heparin protected the two hepatoma cell lines from TpT actions and decreased the rate of TpT induced S phase block and cell death. These results suggest that heparin and HS might interfere with the function of TpT in liver and liver cancer.

## 1. Introduction

Heparin and heparan sulfate (HS) are polysulfated sugars, members of glycosaminoglycans (GAGs), present in animal and human tissue in free or protein bound forms.

Heparan sulfate glycanated proteins are found in the extracellular matrix and on the cell surface [1]. Recent studies provide ample evidence on the central role of these molecules in cell life including cellular organization, cell behavior, and cell signaling [1, 2]. Heparin und heparan sulfates bind several growth factors [3–7], hormones [8], cytokines [6, 9], and chemokines [10, 11] that are implicated in cell regulation [12] in several ways.

The cellular role of HS has been studied for years without a major breakthrough achieved [13–18]. Biochemical approaches failed to collect convincing data for intracellular proteoglycan activity. Recently tentative evidences were provided supporting the regulatory effect of HS on cell proliferation and showing that these GAGs affect DNA-transcription factor interactions [19]. Our previous experiments resulted in similar conclusions [17]. For the first time confocal microscopy evidenced the nuclear localization of GAGs and proteoglycans [20–22]. Since then the nuclear function of proteoglycans is coming to focus of interest [22]. Nevertheless, the issue is still an elusive part of proteoglycan research.

We reported that heparin and liver HS inhibit the plasmid relaxation activity of topoisomerase I enzyme in vitro [21]. Furthermore, we provided evidence for heparin and HS cellular uptake and accumulation in the nucleus [17, 22]. These observations motivated us to investigate if GAG molecules are able to interfere with topoisomerase I (topo I) activity and modify the effect of topo I inhibitory drug topotecan (TpT) [23].

## 2. Materials

### 2.1. Liver Tissue

Surgical specimens from cancer patients were sent to our department for histological diagnosis and were used with the permission of the regional ethical committee. The samples were frozen in liquid nitrogen and stored at −80°C until used.

### 2.2. Cells

American Tissue Type Culture Collection HepG2 and Hep3B cell lines were used after 12–15 passages. Cells were plated at a density of 2 × 10^5^ cells/mL into six-well plates in 2 mL/well Dulbecco's modified Eagle's medium with 5% (v/v) fetal calf serum (GIBCO-BRL).

### 2.3. Chemicals

Unless specified otherwise, the chemicals were purchased from Merck (Darmstadt, Germany). Hind III and Klenow DNA polymerase enzymes were obtained from Promega (Madison, USA). Topotecan was a gift of SmithKline Beecham (King of Prussia, USA). Heparin was purchased from Sigma (Steinheim, Germany).

Protein concentration was determined by using the Coomassie protein assay kit of Pierce (Rockford, USA). Recombinant topo I and polyclonal human anti-topo I IgG (scl-70) from Topogen (Columbus, USA) were used for western blot.

## 3. Methods

### 3.1. Cell Numbers, Viability, and Morphology

Mitochondrial succinate dehydrogenase activity [24] was determined by 3-(4,5-dimethylthiazol-2-yl)-2,5-diphenyltetrazolium bromide (MTT) test, and cell numbers were counted in a hemocytometer.

Morphology of the two hepatoma cell lines was studied either by growing them onto coverslips or by preparing cytospin slides. Cells were visualized with hematoxyline-eosine staining.

### 3.2. Determination of Cell Cycle Parameters

HepG2 and Hep3B cells were washed twice with PBS then suspended in a buffer containing 0.1% sodium citrate, 0.1% Triton X-100, and 0.05 mg/mL ribonuclease, pH 7.7, at 10^6^ cell/mL density. Before the analysis, the cells were stained with 50 *μ*g/mL propidium-iodide (Sigma, Steinheim, Germany).

Cell cycle parameters were measured on a FACScan flow cytometer (Becton Dickinson, San Jose, USA) scanning the propidium-iodide signals and the forward and side scatter parameters. The Multicycle software of Robinovitch (Phoenics Flow San Diego, USA) was used for analyzing the results.

### 3.3. Protein and GAG Isolation, Quantification

Nuclei from liver specimens were isolated on saccharose gradient, according to Hogeboom [25]. The method of Duguet was used when nuclei were isolated from hepatoma cell lines [26]. Isolation was carried out in the presence of 1.0 mM phenyl-methylsulfonyl fluoride (PMSF). Nuclei were further extracted with 0.35 M NaCl buffer as described elsewhere [27]. Protein concentration of the nuclear extract was determined by the Coomassie protein kit of Pierce Biotechnology, Inc. (Rockford, USA) according to the protocol of the manufacturer. The isolation of GAG and HS, their electrophoresis, and their colorimetry were described previously [17, 21, 28].

### 3.4. Topoisomerase I Relaxation Assay

ATP independent relaxation of supercoiled pBR322 plasmid Stratagene (La Jolla, USA) was done in standard 30 *μ*L reaction mixture containing various amounts of 0.35 M NaCI nuclear protein extract, 0.5 *μ*g supercoiled plasmid DNA, 40 mM Tris-HCl, pH 7.5, 100 mM KCl, 10 mM MgCI, 2.5 mM dithiotreitol, 0.5 mM EDTA, 1 mM phenyl-methylsulfonyl fluoride, and 1 *μ*g bovine serum albumin (as carrier protein) [29]. Samples were incubated at 37°C for 30 min with or without various GAGs as indicated. The addition of GAGs did not influence the pH of the reaction mixture. Enzymatic reactions were terminated by adding 1 *μ*L 10% sodium dodecylsulfate and 1 *μ*L (10 mg/mL) proteinase K. Relaxation of supercoiled DNA plasmid was determined by running the samples on analytical 1% agarose gel in Tris-boric acid buffer (50 mM Tris, 5 mM boric acid, 1 mM EDTA, pH 8.1.) for 12 h at 24 V. Gels were poststained with 0.1 *μ*g/mL ethidium bromide and visualized under UV light [29].

#### 3.4.1. Plasmid Cleavage Reaction for Topoisomerase I

pBR322 plasmid was linearized with EcoRI enzyme and then end-labeled with 5 *μ*Ci *α* ^32^ dATP and 20 U Klenow DNA polymerase at 30°C for 15 min. Heating at 75°C for 10 min terminated the reaction. The labeling was removed from one end of the plasmid by Hind III digestion. Unincorporated radioactivity was separated by filtration through a Sephadex G50 (Pharmacia, Uppsala, Sweden) column. Ten microgram protein from the 0.35 M NaCI extract of liver or cancer cell nuclei was coincubated with 100 *μ*M topotecan and 8000 cpm labeled plasmid with or without 2 *μ*M heparin for 10 min. The reaction was terminated with SDS and proteinase as described above and the samples were run on 1% agarose gel with denaturing sample buffer (0.45 M NaOH). The gels were dried and exposed to Kodak X-omat film [29].

#### 3.4.2. Electrophoretic Mobility Shift Assay to Study the Competition of DNA and Heparin for Topoisomerase I

pBluescript plasmid was digested with Hpa II restriction enzyme. One of the restriction fragments with 516 base pair was separated and labeled with DIG-11 dUTP and terminal deoxynucleotide transferase (Roche, Mannheim, Germany), as suggested by the manufacturer. Based on its sequence analysis the labeled fragment contained 8 potential topoisomerase I binding sequences [30]. Ten unit purified topoisomerase I enzyme was incubated with 35 ng digoxigenin labeled DNA fragment in 40 mM Tris-HCl pH 7.5, 100 mM KCl, 10 mM MgCl, 2.5 mM dithiotreitol, and 0.5 mM EDTA. 1 mM phenylmethylsulfonyl fluoride and 1 *μ*g bovine serum albumin (as carrier protein) in a total volume of 20 *μ*L. Identical reaction mixtures were supplemented with 10 or 100 ng commercial heparin. Incubation was carried out at 37°C for 15 min. Subsequently, the samples were run on 1.2% agarose gel and blotted to positively charged nylon membrane (Boehringer (Roche), Mannheim, Germany). The positions of the DNA bands were visualized by sheep alkaline phosphatase-conjugated antidigoxigenin Fab fragments (Roche, Mannheim, Germany), using NBT and BCIP (Roche Applied Science) as chromogens.

#### 3.4.3. Analysis of the Raw Data

The assays have been run in triplicates and statistical significance has been calculated based on data distribution (normal or non-parametric) using a Student's* t*-test or a Mann-Whitney test using Graphpad Prism 4.03 (Graphpad Software Inc., Suite, La Jolla, CA, USA).

## 4. Results 

### 4.1. Influence of Heparin on Topotecan-Induced Cell Growth Retardation

**Figure 1:**
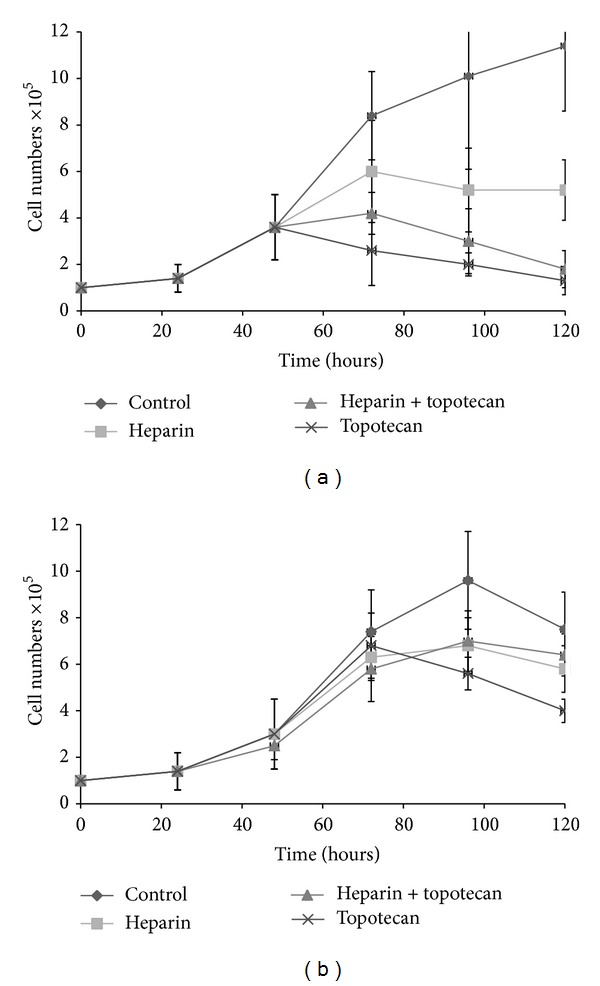
Effects of heparin and TpT on HepG2 (panel a) and Hep3B (panel b) cell numbers after 48–120 h incubation (results of 3 independent experiments). After 48 h of plating, serum has been withdrawn and the action of heparin and TpT was studied under serum-free conditions. Cells were grown for 72 h in the presence of 1 *μ*M TpT, 100 *μ*g/mL heparin alone, or in combination of the two. The curves represent the average of 6 parallels. Cells were counted daily. Both cell lines reached the exponential phase of cell growth around 48 h. Inhibitory action of TpT occurred at 72 h, which was exerted continuously thereafter (difference is significant with Student's* t*-test *P* < 0.001). 100 *μ*g/mL heparin inhibited the growth of both hepatoma cell lines, but at a lower extent than TpT (*P* > 0.05 with Student's* t*-test for HepG2, *P* = 0.02 for Hep3B). In combined treatment heparin rescued the cells against TpT action. The protection was significant for Hep3B cells (b) (*P* < 0.001 with Student's* t*-test), but not for HepG2 cells (Figure 1(a)) (*P* > 0.1).

To assess the interference of heparin with TpT, the two hepatoma cell lines were treated with 1 *μ*M TpT alone or together with 100 *μ*g/mL heparin. The effect was evaluated measuring the growth parameters of untreated or heparin treated cells. After 48 h of plating, the serum has been withdrawn and the action of heparin and topotecan was studied under serum-free conditions. Cells were counted daily. Figures 1(a) and 1(b) show that both cell lines reached the exponential phase of cell growth around 48 h after plating. Inhibitory action of TpT occurred at 72 h and was exerted continuously thereafter (*P* < 0.001 with Student's* t*-test). The growth of both hepatoma cell lines was inhibited by 100 *μ*g/mL heparin, but at a lower extent than by TpT (*P* > 0.05 with Student's* t*-test for HepG2, *P* = 0.02 for Hep3B). A combined treatment revealed that heparin is capable of rescuing the cells against TpT action. This effect was statistically significant in Hep3B cells (*P* < 0.001 with Student's* t*-test), but not in HepG2 cells (*P* > 0.1).

### 4.2. Changes in Cell Cycle Parameters

Although heparin inhibited the proliferation of both hepatoma lines, no changes in cell cycle parameters were discernable. TpT induced dramatic G1-S phase block and cell death in both cell lines. Both effects were reduced when TpT was administered together with heparin (Table 1(a)). After TpT exposure, the ratio of apoptotic cells increased more than three and four times in HepG2 and Hep3B cells, respectively. This level fell back to the original value after the combined heparin + TpT treatment of Hep3B cell line, while only 30% protection was achieved in case of HepG2 cells (Table 1(b)). The S phase block decreased with 13% compared to TpT treatment in both cell lines, which was statistically significant only in Hep3B cells (Table 1(a)).

### 4.3. Topoisomerase I Activity of Liver Specimens

**Figure 2:**
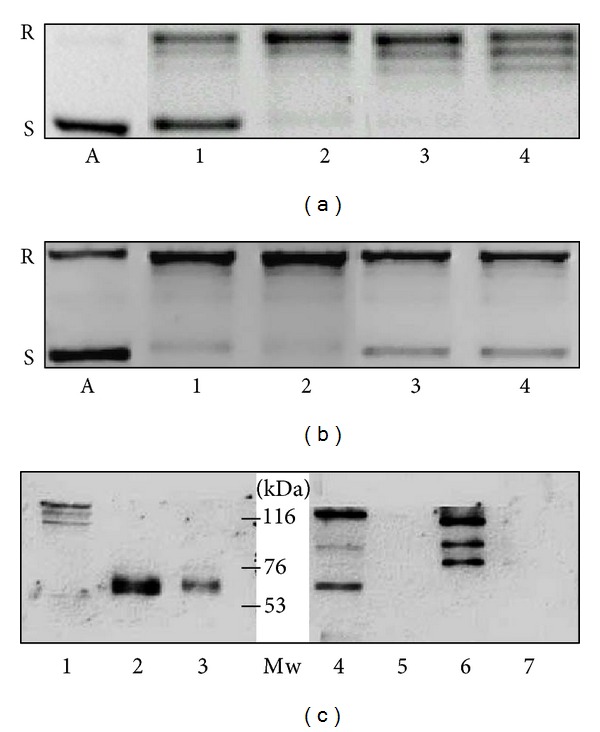
Differences of topoisomerase I activity in liver samples (a) and in hepatoma cells (b) (representative image of three independent experiments). Topoisomerase I activities in liver samples (a) and hepatoma cell lines (b) of 250 and 500 ng nuclear extract from peritumoral liver (a 1, a 2), hepatocellular carcinoma (a 3, a 4), HepG2 (b 1, b 2), and Hep3B cells (b 3, b 4). The activities of the specimens are related to the amounts of topoisomerase I protein in the cell nuclear extracts, as it is demonstrated on a western blot (c). Lane 1: Hep3B, lanes 2 and 3: HepG2, lanes 4 and 6: hepatocellular carcinomas, and lanes 5 and 7: peritumoral liver tissues. In addition to the 120 kDa band of the whole protein, the antibody reacts with more degradation products of the enzyme, including the 67 kDa catalytic fragment. A: plasmid control without cell nuclear extract. R: relaxed, S: supercoiled plasmid.

The topo I enzymatic activities of surgically removed human liver and hepatocellular carcinomas as well as those of two hepatoma cell lines were studied. Nuclear extracts from peritumoral liver specimens showed low topo I activity. In contrast, 200 ng of nuclear protein from liver cancer resulted in total relaxation of the pBR322 plasmid. An identical amount of protein from peritumoral liver left more than half of the plasmid unattached. The activity in HepG2 cells was as high as in the primary liver cancer. Interestingly the less differentiated Hep3B hepatoma cell line retained only moderated topoisomerase I activity (Figures 2(a) and 2(b)).

**Figure 3:**
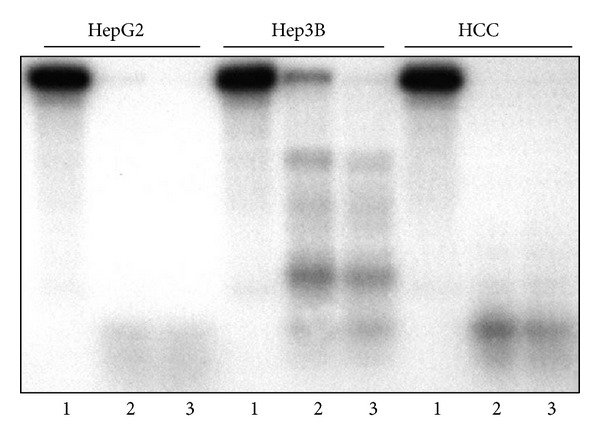
Differences of topoisomerase I plasmid cleavage activity trapped by TpT in liver samples and in hepatoma cells (representative image of three independent experiments). pBR322 plasmid was linearized and end-labeled with 5 *μ*Ci *α* ^32^P ATP by using 20 U Klenow DNA polymerase. To remove the labeling from one end, the linearized plasmid was digested with Hind III restriction endonuclease. Ten microgram 0.35 M NaCI nuclear extracts of HepG2, Hep3B, and human HCC specimens were incubated with 8000 cpm linearized plasmid in the absence or presence of 100 and 200 *μ*M topotecan. Lanes 1: cleavage reaction without topotecan, lane 2: cleavage reaction with 100 *μ*M topotecan, and lane 3: cleavage reaction with 200 *μ*M topotecan. The activity of Hep3B extract was much lower than that of HepG2 and human Iiver cancer.

Western blots loaded with 15 *μ*g of nuclear protein extracts indicated that the measured activities were linearly dependent on the amounts of topoisomerase protein in the cells. A significantly higher amount of topo I protein was detected in HepG2 cells and primary HCC than in the Hep3B cell line. The protein in peritumoral livers was below the detection level (Figure 2(c)). The cleavage reaction of TpT-trapped enzyme corroborated the activity of the liver and tumor samples. When using 10 *μ*g HepG2 and human HCC nuclear extract, a complete fragmentation of the end-labeled plasmid was observed in the presence of 100 *μ*M TpT. The same amount of Hep3B nuclear protein was considerably less effective (Figure 3).

### 4.4. Inhibitory Action of Glycosaminoglycans on Topoisomerase I Activities

Nuclear extracts with high topoisomerase I activity from HepG2 cells and a surgically removed hepatoma were used to assess the inhibitory potential of commercial heparin, normal liver HS, human hepatocellular carcinoma, and peritumoral liver tissue GAG specimens on topo I plasmid relaxation and TpT-trapped cleavage reaction. All GAG specimens but HS from liver carcinoma inhibited the plasmid relaxation assay in a dose-dependent manner. In this measure, commercial heparin was the most effective confirming an earlier report [23].

**Figure 4:**
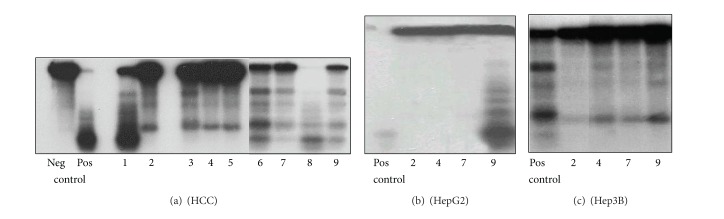
Inhibitory effect of heparin, normal and peritumoral liver heparane sulfate (HS), and hepatocellular carcinoma heparane sulfate on the TpT induced topo I cleavage reaction (representative image of three independent experiments). The origin of nuclear extracts: (a): hepatocellular carcinoma; (b): HepG2 cells; (c): Hep3B cells. The reaction mixture contained pBR322 plasmid DNA, nuclear extracts as described, 100 *μ*M topotecan in all samples as well as in the positive controls. In negative control topotecan was not added. Types and concentrations of GAGs are as follows: (a1): heparin 1 *μ*M; (a, b, c 2): heparin 2 *μ*M; (a3): normal liver HS 1 *μ*M; (a, b, c 4): normal liver HS 2 *μ*M; (a5): normal liver HS 3 *μ*M; (a6): peritumoral HS 1 *μ*M; (a, b, c 7): peritumoral HS 2 *μ*M; (a8): hepatocellular carcinoma HS 1 *μ*M; (a, b, c 9): hepatocellular carcinoma HS 2 *μ*M.

In cleavage reactions, the efficacy of heparin and normal liver HS was identical. Peritumoral liver GAG inhibited the cleavage better than the HCC GAG did (not shown). When this experiment was repeated by using isolated peritumoral and HCC HS, the result was similar (Figure 4) indicating that heparan sulfate is the active GAG component and is responsible for the inhibitory activity. The inhibitory action of GAGs depended on the topoisomerase I activity of the nuclear extract. Even liver cancer HS decreased the TpT-induced cleavage reaction of Hep3B cells (Figure 4).

**Figure 5:**
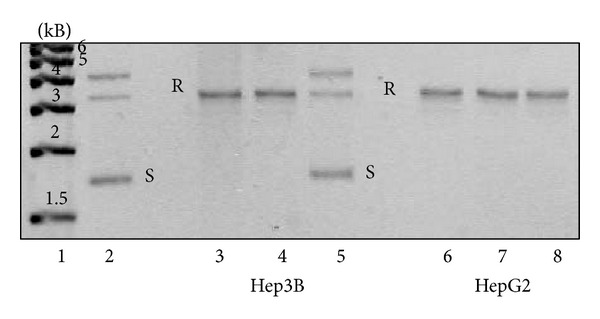
Effect of heparin treatment on topoisomerase I plasmid relaxation activity of HepG2 and Hep3B cells (representative image of three independent experiments). Cells were plated to 6 well plates at 2.5 × 10^5^ cells/plate and grown for 24 h in the presence of 5% fetal calf serum. Subsequently, the serum was replaced with bovine serum albumin (BSA), and the cells were exposed to 100 *μ*g/mL heparin for 24 h (lanes 4 and 7) and 48 h (lanes 5 and 8). Cell nuclear extracts were used for topo I relaxation activity measurements as described. Heparin exposure for 48 hours resulted in total loss of enzyme activity of Hep3B cells (lane 5). Heparin did not affect the activity of HepG2 cells (lanes 4 and 5). Lane 1: DNA size standard, 1 kiloBase (kB) ladder; lane 2: plasmid control without protein (S: superhelix, R: relaxed); lanes 3 and 6: plasmid relaxation in untreated cells.

We also studied if this phenomenon, observed in a cell-free system, can also be detected when cell cultures were treated with heparin. To this end, hepatoma cell lines were treated with 100 *μ*g/mL heparin for 24 and 48 h. Thereafter, nuclear extracts were prepared and used for plasmid relaxation, as it is shown in Figure 5. Cell nuclear extracts of heparin-treated Hep3B cells did not exert topo I activity. Nevertheless, an identical amount of heparin could not inhibit the activity of the enzyme in HepG2 cells.

### 4.5. Competition of Heparin and DNA for Topoisomerase I

**Figure 6:**
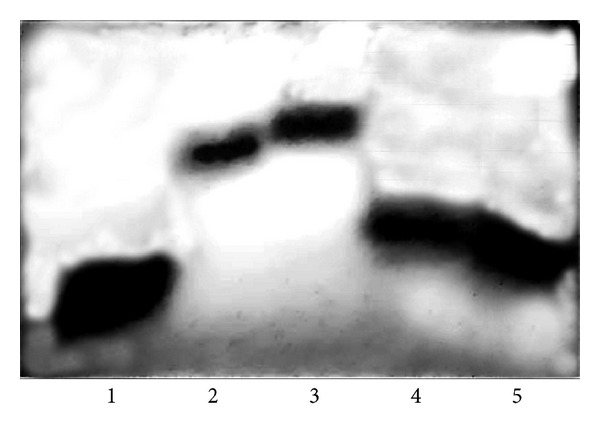
Competition of heparin and DNA for topoisomerase I (representative image of three independent experiments). Effect of heparin on the DNA gel retardation produced by 10 U purified topoisomerase I protein. DNA fragment of 516 base pair size with 8 potential topo I-binding sequences was end-labeled with digoxigenin-UTP. Thirty-five ng DNA was incubated with 10 U topoisomerase I alone or in the presence of 10 and 100 ng heparin. Samples were run or 1% agarose, blotted to nylon membrane, and developed with antidigoxigenin alkaline phosphatase. 1: control DNA, without protein; 2 and 3: DNA with topoisomerase I; 4 and 5: DNA and topoisomerase I, with 10 and 100 ng heparin, respectively.

As topoisomerase I is a heparin-binding protein, we tested if heparin competed with the DNA for the enzyme. Changes in the electrophoretic mobility shift indicated that 10 *μ*g heparin effectively inhibited the mobility shift caused by 10 unit topo I enzyme on 35 ng DNA (Figure 6).

## 5. Discussion

Our previous studies on various human cancer specimens revealed that the increase in the amount of proteoglycans and their sugar components is one of the most striking features of these tumors [31, 32]. As a general rule, we found about tenfold increase of chondroitin sulfate and fivefold increase of heparan sulfate in surgically removed liver and kidney cancer tissues. The biological significance of these changes awaited explanation. The pharmacological effects of heparin have been known for a long time, but contradictory observations were reported on its capacity to inhibit cell proliferation [33–36]. While heparin is mainly present in mast cells, HS that is structurally strongly related to heparin is present everywhere in the living organisms [37]. Earlier we demonstrated that cells could take up labeled heparin and liver HS and transport them into the nucleus [17, 21].

Furthermore, not only GAGs but also proteoglycans have been detected in the nucleus [38]. One possible role of nuclear HS is to shuttle the nuclear transport of heparin-binding growth factors, such as basic fibroblast growth factor (FGF-2). Once in the nucleus, these growth factors might directly modulate cellular activities [39]. A less-known way of regulation of this process is through heparanase enzyme, an endoglycosidase, implicated in cancer progression and metastasis [40, 41]. Heparanase can be localized to both the plasma membrane and the nucleus, and thus its interference with action of heparin is conceivable. Both its localization and cellular levels are finely regulated [42].

The evidences for the regulatory significance of heparin and HS justify our efforts to look for physiological or pathological cell nuclear functions where heparan sulfates could be involved [43]. We addressed the question if heparin induced inhibition of cell proliferation might be related to its ability to bind and inactivate nuclear proteins. We focused on topoisomerase I. Certainly, heparin and liver HS, but not liver cancer HS, bound and inhibited topoisomerase I plasmid relaxation in vitro [21, 44]. Our present work also demonstrates that heparin and HS hinder the in vitro plasmid cleavage effect of TpT. This effect was much more obvious when cell nuclear extracts were obtained from liver tumor specimens with low or moderate topoisomerase I activity. This in vitro phenomenon seemed to be important from two points of view. First, heparin or HS could protect normal surrounding tissues with low topoisomerase I activity from the cytotoxic action of TpT. Second, however, the same mechanism could rescue tumors with low or modest topoisomerase I activity from TpT.

The HepG2 cell line with high and the Hep3B cell line with moderate enzymatic activity served as a model to test this hypothesis. In support of the results obtained in a cell-free system, heparin exposure abolished the moderate topoisomerase I activity of the Hep3B cell line, while HepG2 cells retained their enzymatic activities. As the efficacy of TpT depends on the actual activity of topoisomerase I, it was reasonable to expect that, if administered together, heparin will interfere with the action of the drug. Certainly, using the Hep3B cell line with low topoisomerase I activity, the growth inhibitory effect of TpT decreased in the presence of heparin, while only a modest, transient heparin protection has been achieved on the HepG2 cell line. However, the mechanism of action was still a question.

Heparin alone did not affect the cell cycle. More likely, its protective effects against TpT action were related to its topo I binding capacity. In an assay mixture containing heparin, recombinant topo I and labeled DNA heparin appeared to compete with DNA for the binding of topo I. Thus, in the presence of heparin or HS a lower proportion of topoisomerase I could be bound covalently to DNA by TpT, thus preventing DNA fragmentation. The binding capacity and the amount of heparin or heparan sulfate can determine the proportion of topoisomerase I that will not interact with DNA. The decrease in dead cell fraction after a combined TpT-heparin treatment provided further support to this hypothesis.

Our results are in a good agreement with those clinical observations that aimed to treat liver tumors with TpT. Similarly to the HepG2 cell line, hepatoblastomas respond well for TpT treatment [45, 46], whereas the drug efficacy on liver cancers is modest at best [47]. In the general practice TpT is administered without knowing the topoisomerase I activity status of the tumors. Even though additional studies are warranted on the subject, our current results suggest that for maximum efficacy treatment regimens should avoid concomitant application of heparin and a topoisomerase inhibitor. Having said this, as HS of the tumor itself appears to be ineffective to inhibit topoisomerase I activity, it does not reduce the efficacy of TpT, at least not in the case of liver carcinomas.

## Figures and Tables

**(a):** 

% of cells	HepG2			Hep3B		
Treatment	G_1_	S	G_2_	G_1_	S	G_2_
Control	52,6 ± 4,08	35,7 ± 3,29	11,6 ± 2,19	78,7 ± 0,42	15,9 ± 0,14	5,5 ± 0,28
1 *µ*M TpT	15,8 ± 1,3^1^	80,8 ± 15,5^1^	4,2 ± 0,07^1^	2,06 ± 0,07^1^	65,6 ± 0,99^1^	13,7 ± 1,06^1^
100 *µ*g/mL Heparin	58,9 ± 1,39	28,3 ± 3,58	12,8 ± 0,59	81,1 ± 0,28	14 ± 0,28	5 ± 0,07
Hp + TpT	14,5 ± 0,45	70,2 ± 3,97^3^	15,2 ± 0,68	35,4 ± 0,1	56,4 ± 2,33^2^	8,1 ± 2,4

^1^Student's *t*-test: *P* < 0.02 for HepG2, *P* < 0.005 for Hep3B (TpT compared to control).

^2^Student's *t*-test: *P* < 0.005 (Hp + TpT compared to TpT alone).

^3^Student's *t*-test: *P* > 0.1 (Hp + TpT compared to TpT alone).

**(b):** 

%-of cells in apoptotic gate	HepG2	Hep3B
Control	5,65 ± 0,595	**3,53 ± 0,93**
1 *µ*M TpT	18,67 ± 3,53^1^	16, 49 ± 0, 821^1^
100 *µ*g/mL heparin	9,06 ± 0,16^2^	**2,48 ± 0,22**
Hp + TpT	12, 69 ± 2, 34^3^	4,44 ± 1, 07^3^

^1^Student's *t*-test: *P* < 0,001 (TpT compared to control).

^2^Student's *t*-test: *P* < 0,05 (Hp compared to control).

^3^Student's *t*-test: *P* < 0,05 (Hp + TpT compared to TpT alone).
